# Re-amputation in patients with diabetes-related minor amputations who underwent physical therapy during their hospitalization

**DOI:** 10.1186/s13047-021-00454-y

**Published:** 2021-02-17

**Authors:** Shinsuke Imaoka, Koji Sato, Masahide Furukawa, Minoru Okita, Toshio Higashi

**Affiliations:** 1 Department of Rehabilitation, Oita Oka Hospital, 3-7-11, Nishitsurusaki, Oita City, Oita Prefecture 870-0192 Japan; 2 Unit of Medical Sciences, Nagasaki University Graduate School of Biomedical Sciences, 1-7-1 Sakamoto, Nagasaki City, Nagasaki Prefecture 852-8520 Japan; 3 Department of Plastic Surgery, Oita Oka Hospital, 3-7-11, Nishitsurusaki, Oita City, Oita Prefecture 870-0192 Japan

**Keywords:** Diabetes mellitus, Re-amputation, Physical therapy, Amputation, Minor amputation

## Abstract

**Background:**

Diabetes-related foot lesions are a major cause of non-traumatic lower limb amputations and are associated with a high re-amputation rate. Lesions can cause hindrance in activities of daily living, reduce physical function, and lower a patient’s quality of life. Physical therapy is necessary to prevent these limitations. Thus far, there has been limited investigation into the re-amputation rate in patients who have undergone physical therapy. This study aimed to elucidate modifiable risk factors for re-amputation in patients with minor amputations who were treated with physical therapy during their hospitalization.

**Methods:**

This was a retrospective cohort study of 245 consecutive hospitalized patients who presented to our Wound Care Center between January 2015 and February 2018 and received physical therapy after a minor amputation. Participants were identified from admission records to surgical and physical therapy units stored in the electronic medical records. We examined re-amputations that occurred in the ipsilateral lower extremity during the 1-year post-discharge outpatient period. The maximum follow-up period was set at 1 year. We used Cox proportional hazards analysis to examine factors affecting the risk of re-amputation.

**Results:**

Of the 129 patients enrolled, 42 patients (32.5%) underwent re-amputations during an average observation period of 6.2 months (range, 2.1 to 10.9 months). The factors associated with re-amputation were a requirement for hemodialysis, ankle dorsiflexion angle, and the Functional Independence Measure (FIM) ambulation score.

**Conclusions:**

In diabetes patients with minor amputations, a requirement for hemodialysis, ankle dorsiflexion angle, and the FIM ambulation score were shown to be modifiable risk factors for re-amputation. This emphasizes that maintaining vascular endothelial function through lower limb muscle exercises for hemodialysis, improving ankle mobility, and relieving plantar pressure during walking are necessary to reduce the risk of re-amputation. Patients with these risk factors should be encouraged to participate in physical therapy.

## Background

In a survey of lower limb amputations in Japan, the amputation rate in the 1960s was 1.6/100,000 patients, and 70% of the amputations were caused by trauma; however, in the 2000s, the amputation rate was reported to be 5.8/100,000 patients, and the cause was peripheral circulatory disturbances in 66.2% of cases [1]. A prognostic study of amputee patients reported that the mean age of patients with foot amputations was 72.4 years and that the re-amputation rate was 18.2% [2].

A report examining the prognosis after minor amputation reported that 31.5% of patients required re-amputation within 2 years [3]. These reports indicate that amputees are older and have a higher short-term re-amputation rate.

After minor amputation, a patients’ quality of life is reduced, with limitations on their daily activities [4]. In addition, metabolic functions and microcirculatory systems are often impaired [5, 6], and postoperative rest can cause a disuse syndrome, such as lower limb muscle atrophy and reduced physical endurance, leading to a decreased walking ability and limitation in social life. With the decline in walking ability, nursing care may be required for daily life, which may increase social security costs.

In addition, plantar pressure relief during walking and maintaining ankle range of motion (ROM) plays an important role in preventing ulcers that may be a precursor for amputation [7, 8]. Previous studies have shown that the risk factors for re-amputation after minor amputation cases are age [9], wound depth [10], history of peripheral arterial disease [11], and wound infection [12].

Physical therapy may be required to prevent a decline in physical function, activities of daily living, and quality of life. However, there has been limited investigation into re-amputation in patients who have required physical therapy. Therefore, the present study aimed to elucidate modifiable risk factors of re-amputation in patients with a history of minor amputations who received physical therapy during their hospitalization.

## Methods

### Study design and participants

This single-center retrospective cohort study was conducted in the Wound Care Center of Oita Oka Hospital, a community medical support hospital with a multidisciplinary foot care team.

A total of 245 consecutive inpatients who presented to our Wound Care Center between January 2015 and February 2018 and who received physical therapy after revascularization and a minor amputation were included. Participants were identified using information from the admission records (to surgical and physical therapy units) stored electronically. We examined re-amputations in the ipsilateral lower extremity during the 1-year post-discharge outpatient care period.

In this study, a diabetes-related foot was defined as a plantar ulcer associated with neuropathy and peripheral artery disease in patients with diabetes [13]. The amputation region was defined as a minor amputation of the toes, rays, and metatarsal bones. Amputation below and above the knee was defined as major amputation [14]. We excluded patients with the following: (1) infection after minor amputation, (2) major amputation (below and above the knee), (3) death following discharge due to systemic complications, (4) use of a wheelchair for mobility before admission, (5) severe progression of dementia, (6) missing data, and (7) patients who did not visit the hospital for regular outpatient visits (1, 3, 6, or 12 months) after discharge. The reason for excluding patients with postoperative infections was that if an obvious wound infection appeared postoperatively, the rehearsal intervention was discontinued in view of the spread of infection. Patients who had difficulty in undergoing continuous physical therapy due to infection were excluded.

**Fig. 1:**
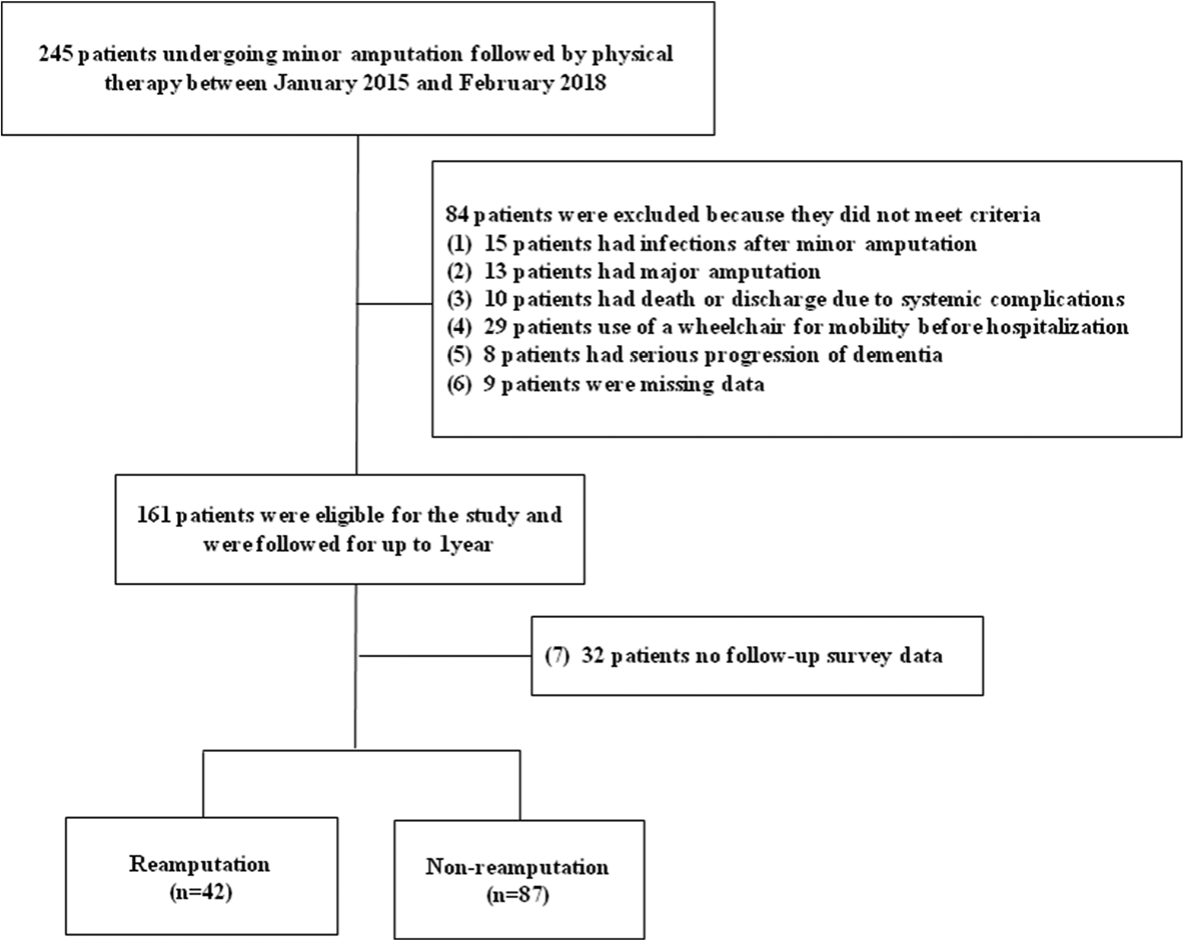
Flowchart of patient selection

The date of death within 1 year after the minor amputation was confirmed by the medical information from the cooperating medical institutions. Finally, 129 patients were enrolled in this study (Fig. 1).

### Data collection and definition

We collected basic data on all patients by reviewing electronic medical records and structured interviews that were conducted when they were admitted for the first amputation. The structured interview involved questions on age, sex, current medical history, cognitive functioning, pre-hospitalization living conditions, and mobility. Measurement items included participants’ basic and medical information, including physical function. Basic information included age, sex, body mass index (BMI), hospitalization days, physical therapy duration, the average length of daily physical therapy in minutes, non-weight-bearing duration, comorbidities (hypertension, heart disease, cerebrovascular disease, and chronic obstructive pulmonary disease), and the requirement (or no requirement) for hemodialysis.

The medical information included laboratory parameters including serum albumin, serum hemoglobin, blood glucose, C-reactive protein, white blood cell counts, and estimated glomerular filtration rate [15].

For data collection, we divided the patients into four groups as follows: The estimated eGFR (1) ≥60 mL/min/1.73 m^2^, (2) 45–59.9 mL/min/1.73 m^2^, (3) 30–44.9 mL/min/1.73 m^2^, and (4) < 30 mL/min/1.73 m^2^. Lower limb blood flow data (skin perfusion pressure and ankle-brachial pressure index), Wound, Ischemia, foot Infection (WIfI) classification system [16], amputation region (toe, ray, and transmetatarsal) [14], foot deformity (Charcot’s joint [17], hallux valgus [18], hammer toe [19], and claw toe [20]). The deformity was determined by experienced plastic and orthopedic surgeons specializing in treating diabetes-related foot lesions based on X-ray images and clinical indicators.

X-ray radiographs were taken periodically before surgery and at 1, 3, 6, and 12 months postoperatively. As the only hospital specializing in podiatry in the prefecture, we regularly perform imaging evaluations as part of our regular practice. Based on the imaging findings, a multidisciplinary wound care team conference is held to evaluate the treatment strategy and degree of progression of the deformity. Physical function was determined by knee extension muscle strength [21], ROM in the ankle joint [22], presence or absence of plantar sensory disorder [23], and ambulation status were evaluated using the Functional Independence Measure (FIM) movement parameter ambulation score [24]. The measurement methods and definitions of the study items are shown in Table 1.

**Table 1:** Definition of variables and factors

Variable	Definition
Body mass index	Calculated by dividing the body weight (kg) by the square of the height (m) and was reported in kg/m².
Hospitalization days	The duration of a single episode of hospitalization. Inpatient days are calculated by subtracting day of admission from day of discharge.
Physical therapy period	Number of days of physical therapy during hospitalization.
Average length of daily physical therapy	Calculated by dividing total hours of physical therapy by the number of days in the hospital.
Non-weight-bearing period	Number of days from the date of surgery to the start of weight-bearing on the lower limb operated on. All patients were eligible, and the initiation of weight-bearing was performed by a physical therapist under the direction of a specialist.
Heart disease	Defined as angina pectoris, myocardial infarction, percutaneous coronary intervention, or coronary artery bypass grafting.
Cerebrovascular disease	Defined as a transient ischemic attack, cerebral infarction, or cerebral hemorrhage.
Amputation region [14]	Toe, ray, and transmetatarsal amputations were defined as minor. Amputation below and above the knee was defined as major.
Estimated glomerular filtration rate [15]	The estimated glomerular filtration rate (eGFR) was calculated based on the new Japanese coefficient Modification of Diet in Renal Disease study equation: eGFR (mL/min/1.73 m²) = 194 × (serum creatinine) − 1.094 × (age) − 0.287 (× 0.739 for females).
Skin perfusion pressure	Blood flow was measured and evaluated using a Laser Doppler (SensiLase PAD4000, KanekaMedix, Osaka, Japan).
Ankle-brachial pressure index	Measured with an automated oscillometric device provided by Omron Colin Co., Ltd. (Tokyo, Japan).
WIfI classification system [16]	WIfI classification system was developed in 2013 and provides an objective classification for wound healing and limb amputation based on three independent risk factors (wound extent, degree of ischemia, and extent of foot infection).
Charcot's joint [17]	Defined as a dislocation of the second metatarsal joint of the tarsus with a rupture of the first metatarsal line and a decrease in the angle of calcaneal inclination.
Hallux valgus [18]	Defined as angulation of the big toe at the first metatarsophalangeal joint > 20°.
Hammer toe [19]	Defined by flexion deformity at the PIP joint with extension at the DIP joint and a neutral or extended position of the MTP joint.
Claw toe [20]	Defined as an extended MTPJ, flexed PIPJ, flexed DIPJ, and determined via clinical examination.
Knee extension muscle strength [21]	Maximum voluntary isometric knee extension muscle strength was measured using a hand-held dynamometer (μ-tasF-1, Anima, Tokyo, Japan). For knee extensor strength measurements, participants were asked to sit on a chair with the knee flexed at 90° and push at maximum strength against the dynamometer pad for 5 s. Isometric knee extensor strength was measured twice per side, and the highest value for the right and left legs was used to represent the knee extensor muscle strength.
Range of motion (ankle joint) [22]	The ankle joint was examined in the neutral position, with the patient supine; a vertical line is marked on the patient’s skin from the heel to midcalf, and the maximum range of dorsiflexion in passive motion is measured in degrees with a goniometer.
Plantar sensory disorder [23]	The presence or absence of plantar sensory impairment was considered as neuropathy when the evaluator size of the Semmes-Weinstein-monofilament was 5.07.We performed three-site tests involving the plantar aspects of the great toe, third metatarsal, and fifth metatarsal.
Functional Independence Measure (ambulation) [24]	FIM is an 18-item, clinician-reported scale that assesses function in six areas including self-care, continence, mobility, transfers, communication, and cognition. Each of the 18 items is graded on a scale of 1–7 based on level of independence in that item (1 = total assistance required, 7 = complete independence). Ambulation items were rated according to the amount of assistance from 1 point (total assistance: able to walk less than 15 m) to 7 points (full independence: able to walk 50 m and move with no assistance).

### Main study outcome

In this study, all patients with minor amputations were followed up, with data collected from electronic medical records, for 1 year after surgery or until death. The endpoint was the presence or absence of re-amputation within 1 year after surgery. Re-amputation was defined as an amputation on the same side of the limb as the initial amputation. To detect the presence or absence of re-amputation, the operative information in the electronic medical record was checked; in addition, the date of amputation and the site of surgery were identified. Our Wound Care Center is the only facility of its kind in the prefecture; therefore, post-discharge outpatient follow-up is basically limited to our facility. As a rule, outpatient visits to the hospital are conducted at intervals of 1, 3, 6, and 12 months after discharge.

### Physical therapy program

Physical therapy was provided to patients to improve their physical function and walking ability. The first postoperative day started with strength training and ROM exercises of the hip and knee joints, which were performed according to the level of pain experienced by the patient. Additionally, a walking practice started after wound healing. The physiotherapy session and physical function measurements were performed by two experienced staff physiotherapists.

### Statistical analysis

Mann-Whitney U-test, t-test, and χ2 test were used to compare background characteristics and indices of physical function between the two groups with re-amputation histories versus groups with no re-amputation histories, depending on the data characteristics. Multivariate Cox regression analysis was also performed after adjusting for confounders by inputting sex [2], age [10], serum albumin levels [25], and knee extension muscle strength [26] as covariates with reference to items that were significant in univariate analysis and previous studies to identify factors associated with re-amputation. To account for multicollinearity in this process, variables considered clinically significant were left in the model if the absolute value of the correlation coefficients between the independent variables was greater than 0.7. In addition, incomplete data sets (missing data) were excluded from the multivariate Cox regression analysis for case-pair-wise deletions.

The incidence of the presence or absence of re-amputation was calculated using Kaplan-Meier curves for the extracted factors. Differences between groups were estimated using the log-rank test. All statistical analyses were performed using R version 3.2.5 (R Foundation for Statistics Computing, Vienna, Austria). The significance level was set to *P* < 0.05.

## Results

**Fig. 2:**
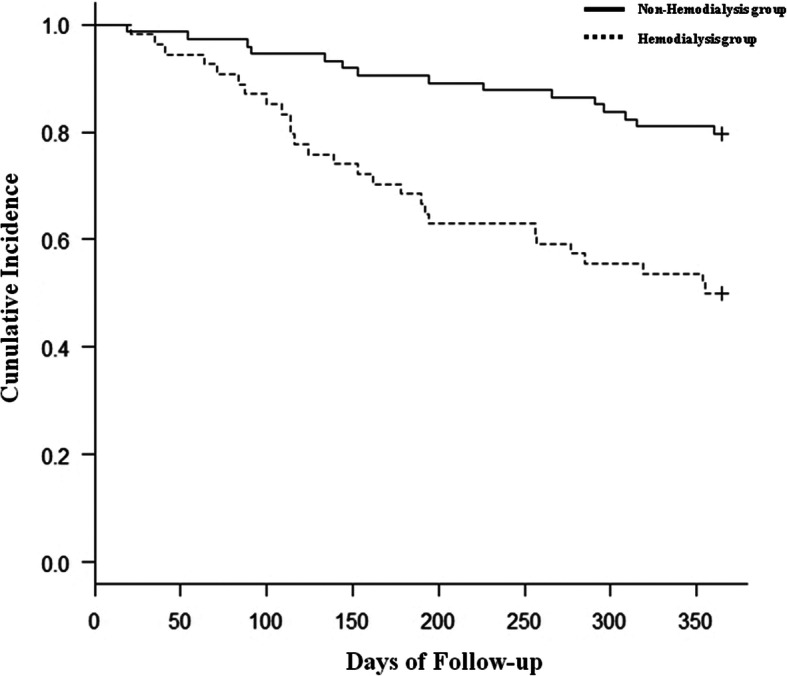
Kaplan-Meier curves of survival versus re-amputation: presence or absence of hemodialysis

**Fig. 3:**
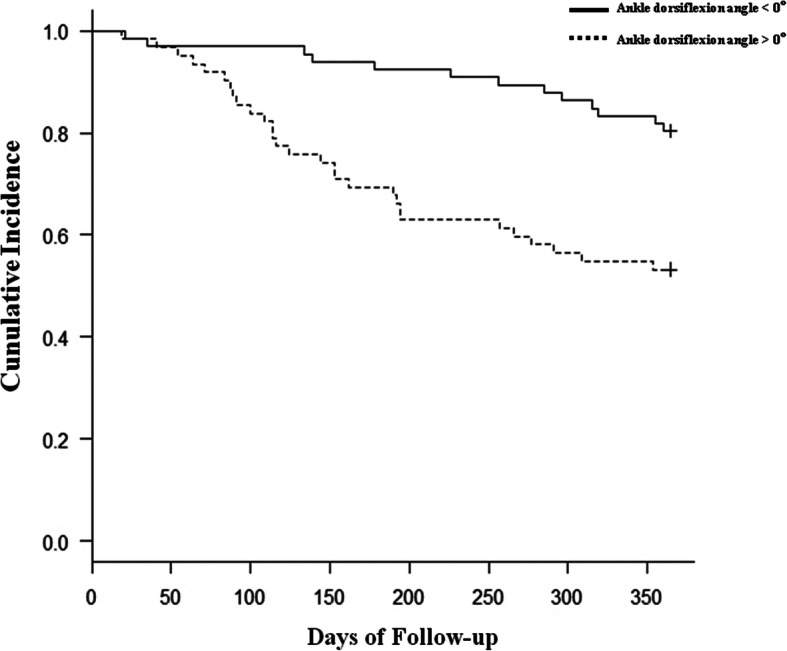
Kaplan-Meier curves of survival versus re-amputation: presence or absence of ankle dorsiflexion limitation

**Fig. 4:**
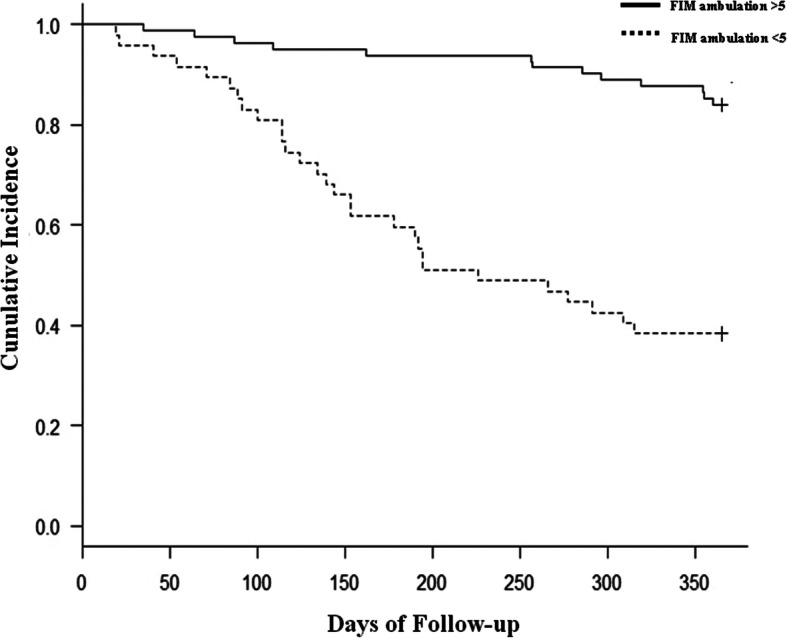
Kaplan-Meier curves of survival versus re-amputation: ambulation group and non-ambulation group

Of the 129 patients enrolled, 42 (32.5%) underwent re-amputation during an average observation period of 6.2 months (range, 2.1 to 10.9 months). Demographic and medical information of the patients is shown in Table 2. The re-amputation group exhibited significantly higher rates of hemodialysis and the FIM ambulation score than the no re-amputation group. Patients in the no re-amputation group demonstrated better ankle dorsiflexion ROM. Univariate Cox regression analysis showed that hemodialysis, ankle dorsiflexion angle, and the FIM ambulation score were potential risk factors for re-amputation. Subsequently, multivariate Cox regression analysis adjusted for age, sex, serum albumin level, and knee extension muscle strength as covariates showed that hemodialysis (HR 2.20, 95% CI 1.12–4.34), ankle dorsiflexion angle (HR 5.82, 95% CI 2.93–11.58), and the FIM ambulation score (HR 3.85, 95% CI 2.00–7.39) were identified as significant risk factors for re-amputation (Table 3). The Kaplan-Meier curves illustrated in Figs. 2, 3, and 4 show the cumulative incidence of re-amputation after minor amputation. Survival analysis using Kaplan-Meier log-rank test showed that the requirement for hemodialysis (Fig. 2), ankle dorsiflexion angle (Fig. 3), and the FIM ambulation score (Fig. 4) were significantly associated with survival (*P* < 0.05).

**Table 2:** Patients categorized into re-amputation and non-reamputation

Characteristics	Total (*n*=129)	Re-amputation group (*n* =42)	Non-reamputation group (*n* =87)	*p* value
Age (years)	66.2 ± 12.3	66.7 ± 12.6	66.0 ± 12.3	0.75
Sex, male, n (%)	83 (64.3)	30 (71.4)	53 (60.9)	0.18
BMI (kg/m2)	22.9 ± 2.3	22.7 ± 2.8	23.0 ± 2.5	0.69
Hospitalization days	33.4 ± 15.7	36.2 ± 16.5	34.3 ± 18.1	0.41
Physical therapy period (days)	23.0 ± 9.2	22.6 ± 9.3	23.1 ± 9.2	0.79
Length of daily physical therapy (min)	40.2 ± 14.8	41.5 ± 7.8	39.6 ± 10.3	0.63
Non-weight-bearing period (days)	15(11–20)	15 (11–20)	17 (12–20)	0.71
**Comorbidities, n (%)**				
Hypertension	64 (49.6)	22 (52.3)	42 (48.2)	0.53
Heart disease	40 (31.0)	12 (28.5)	28 (32.1)	0.65
Cerebrovascular disease	6 (0.4)	2 (4.2)	4 (4.5)	0.41
COPD	4 (0.2)	1 (2.1)	3 (3.4)	0.98
Hemodialysis	41 (31.7)	19 (40.4)	22 (25.2)	<0.01
**Laboratory parameters**				
Serum albumin (g/dL)	2.7 ± 1.1	2.6 ± 1.0	2.8 ± 1.1	0.52
Serum hemoglobin (g/dL)	11.4 ± 2.2	11.1 ± 1.9	11.9 ± 2.3	0.09
Blood glucose	183.6 ± 19.3	179.7 ± 17.4	187.2 ± 21.8	0.19
CRP ( mg/dl)	0.4 ± 1.1	0.5 ± 1.4	0.7 ± 0.9	0.35
WBC 103/μL	9435.9 ± 3203.6	9154.7 ± 3069.3	9573.2 ± 3276.0	0.44
**eGFR ( mL/min/1.73 m2) , n (%)**				
eGFR ≥60	39 (30.2)	12 (28.5)	27 (31.0)	0.91
eGFR 45–59	14 (10.8)	4 (9.5)	10 (11.4)	
eGFR 30–44	17 (13.1)	5 (11.9)	12 (13.7)	
eGFR < 30	59 (45.7)	21 (50.0)	38 (43.6)	
**Lower l imb blood flow**				
SPP (mmHg)	36.7 ± 14.6	35.9 ± 14.2	37.1 ± 14.8	0.58
ABI	0.5 ± 0.2	0.6 ± 0.2	0.5 ± 0.3	0.15
**WIfI classification, n (%)**				
W0	23 (17.8)	8 (19.0)	19 (21.8)	0.81
W1	35 (27.1)	13 (30.9)	27 (31.0)	
W2	41 (31.7)	11 (26.1)	19 (21.8)	
W3	46 (35.6)	10 (23.8)	22 (25.2)	
**Amputation region, n (%)**				
Toe amputation	63 (48.8)	22 (52.3)	41 (47.1)	0.06
Ray amputation	42 (32.5)	13 (30.9)	29 (33.3)	0.51
Transmetatarsal amputation	24 (18.6)	7 (16.6)	17 (19.5)	0.34
**Foot deformation, n (%)**				
Charcot's joint	12 (9.3)	4 (8.5)	8 ( 9.1)	0.97
Hallux valgus	19 (14.7)	7 (14.8)	12 (13.7)	0.68
Hammer toe	17 (13.1)	7 (14.8)	10 (11.4)	0.24
Claw toe	19 (14.7)	8 (17.0)	11 (12.6)	0.35
**Physical function**				
Knee Extension Muscle Strength (kef)	20.4 ± 6.1	19.2 ± 5.8	21.0 ± 6.2	0.11
Ankle dorsiflexion angle (°)	6.4 ± 8.5	2.6 ± 9.2	8.3 ± 7.6	<0.01
Ankle plantar flexion angle (°)	17.5 ± 11.5	16.7 ± 11.9	17.8 ± 11.7	0.61
Plantar Sensory disorder, n (%)	94 (72.8)	32 (76.1)	62 (71.2)	0.62
FIM ambulation score	5 (4–6)	6 (5–7)	5 (4–6)	<0.01

Data expressed as mean ± standard deviation, median (interquartile range), n (%). *BMI* Body mass index, *COPD* Chronic obstructive pulmonary disease, *CRP* C-reactive protein, *WBC* White blood cell, *SPP* Skin perfusion pressure, *ABI* Ankle brachial pressure index, *FIM* Functional Independence Measure

**Table 3:** Factors associated with reamputation

Parameter	Univariate analysis			Multivariate analysis		
	HR	95%CI	*p* value	HR	95%CI	*p* value
Age	0.91	0.49-1.69	0.773			
Sex, male	1.005	0.98 -1.03	0.672			
Serum albumin	0.922	0.70 -1.20	0.549			
Knee Extension Muscle Strength	0.949	0.90-0.99	0.031	0.95	0.91-1.00	0.06
Hemodialysis	2.974	1.54-5.73	<0.001	2.2	1.12-4.35	0.02
Ankle dorsiflexion angle	5.572	2.88-10.77	<0.001	5.82	2.93-11.58	<0.001
FIM ambulation score	3.168	1.68 -5.96	<0.001	3.85	2.00-7.39	<0.001

*FIM* Functional Independence Measure, *HR* Hazard Ratio, *CI* Confidence Interval

Influential factors to reamputation (in the first 1 year) the results of Cox proportional hazard analysis

## Discussion

The present study examined factors that influence re-amputation within 1 year of discharge in patients who had undergone minor amputations. We revealed that the requirement for hemodialysis, ankle dorsiflexion angle, and the FIM ambulation score were associated with re-amputation in this patient population significantly (*P* < 0.05). The re-amputation rate was 32.5% within 1 year of discharge, similar to previous findings [27].

Regarding the relationship between hemodialysis and re-amputation, it has been reported that hemodialysis caused periodic fluid fluctuations and worsened microcirculation, thereby promoting blood circulation disorders [28]. Miyajima et al. reported that hemodialysis was also an independent risk factor of major limb amputation [29]. Okamoto et al. reported that approximately 40% of 140 patients who had undergone hemodialysis had peripheral arterial disease [30]. These findings were similar to this study. We, therefore, determined that patients who had undergone hemodialysis may have undergone re-amputation due to peripheral arterial disease. However, it has been reported that diabetes patients on dialysis often have severe calcification of central arteries; thus, accurate ABI values may not be obtained, and they pseudo-normalize [31]. Therefore, we believe that the risk of peripheral vascular disease may be underestimated in the results of this study.

Regarding the association between ankle dorsiflexion angle and re-amputation, Fernando reported that the incidence of ulcers in patients with diabetes, who had limited joint ROM, was as high as 65%, compared to 5% of those with an unrestricted ROM [32]. Lavery et al. reported that minor amputations are a risk factor for ulcer recurrence [33]. In the present study, the recurrence of ulceration after a minor amputation may have led to re-amputation.

Furthermore, Eduardo et al. reported that patients with diabetes who had minor amputations had an average ankle dorsiflexion angle of 9.6° [34]. The mean dorsiflexion angle in the re-amputation group in this study was 2.6°, which was very low compared to that reported in previous studies.

In this study, all patients received foot ROM training early after surgery, and the physical therapy duration was similar for both groups. It has been suggested that regular screening for preoperative range of motion limitations and postoperative ROM practice time should be expanded since interventions after surgery may not improve foot mobility sufficiently.

Secondly, about the association between the FIM ambulation score and re-amputation, the re-amputation group had a higher FIM ambulation score. The re-amputation group had a median FIM ambulation score of 6 and were able to ambulate independently to 50 m using walking aids. Therefore, post-discharge mobility is assessed mainly by walking distance, which may increase following re-amputation compared to that observed in the no re-amputation group.

In addition, a study reported that an increase in cumulative plantar tissue stress associated with the extent of walking distance resulted in wound formation [35]. Therefore, this report suggests that walking with a limited ankle ROM may lead to an increase in cumulative plantar tissue stress. Finally, this finding suggests that physical therapy after minor amputations should incorporate programs that maintaining vascular endothelial function through lower limb muscle exercises for hemodialysis and include activities that maintain ankle mobility. There is also a need to teach adequate plantar pressure relief methods during walking if there is a high risk of revision surgery.

There are several limitations to this study. First, our results were obtained from a single institution. Similar findings derived from other facilities are needed to validate ours for generalizability. Second, the definition of re-amputation was limited to the original hospital only. Thirdly, it was not possible to evaluate the foot pressure. Further, patients who did not receive physical therapy were excluded. In addition, we did not investigate the living conditions and self-management status of patients after discharge from the hospital.

Moreover, the results may not be generalizable to all hospitalized patients with minor amputations; thus, further longitudinal studies with larger samples in multiple hospital settings are required to investigate the re-amputation rates in hospitalized patients with minor amputations.

## Conclusions

Diabetes patients with minor amputations, a requirement for hemodialysis, ankle dorsiflexion angle, and the FIM ambulation score were shown to be modifiable risk factors of re-amputation. This highlights that maintaining vascular endothelial function through lower limb muscle exercises for hemodialysis, improving ankle mobility, and relieving plantar pressure during walking is necessary to reduce the risk of re-amputation. Patients with these risk factors should be encouraged to participate in physical therapy. Further studies with larger samples are needed to confirm our results.

## Abbreviations

BMI: Body mass index
eGFR: Estimated glomerular filtration rate
FIM: Functional Independence Measure
ROM: Range of motion
HR: Hazard ratio
WIfI: Wound, Ischemia, foot Infection
